# Supplementary material for: A Critical View of the Application of the APEX Software (Aqueous Photochemistry of Environmentally-Occurring Xenobiotics) to Predict Photoreaction Kinetics in Surface Freshwaters
Source: Molecules. 2019 Dec 18;25(1):9. doi: 10.3390/molecules25010009 (PMC7017383; doi:10.3390/molecules25010009)
Supplement: Supplementary file 1 [file molecules-25-00009-s001.zip › 3.2.4_gcc-4.4.0.zip!/3.2.4_gcc-4.4.0/3.2.4_gcc-4.4.0/doc/octave/html/interpreter/Complex-Arithmetic.html]

Complex Arithmetic - Untitled


Next: Trigonometry,
Previous: Exponents and Logarithms,
Up: Arithmetic


---

### 17.2 Complex Arithmetic

In the descriptions of the following functions,
z is the complex number x + iy, where i is
defined as `sqrt (-1)`.

— Mapping Function: **abs** (z)  
> Compute the magnitude of z, defined as
> |z| = `sqrt (x^2 + y^2)`.
>
> For example,
>
> ```
>           abs (3 + 4i)
>                => 5
> ```

— Mapping Function: **arg** (z)  
— Mapping Function: **angle** (z)  
> Compute the argument of z, defined as,
> theta = `atan2 (`y`,` x`)`,
> in radians.
>
> For example,
>
> ```
>           arg (3 + 4i)
>                => 0.92730
> ```

— Mapping Function: **conj** (z)  
> Return the complex conjugate of z, defined as
> `conj (`z`)` = x - iy.
>
>
>
> **See also:** real, imag.

— Function File: **cplxpair** (z)  
— Function File: **cplxpair** (z, tol)  
— Function File: **cplxpair** (z, tol, dim)  
> Sort the numbers z into complex conjugate pairs ordered by
> increasing real part. Place the negative imaginary complex number
> first within each pair. Place all the real numbers (those with
> `abs (imag (`z`) /` z`) <` tol`)`) after the
> complex pairs.
>
> If tol is unspecified the default value is 100\*`eps`.
>
> By default the complex pairs are sorted along the first non-singleton
> dimension of z. If dim is specified, then the complex
> pairs are sorted along this dimension.
>
> Signal an error if some complex numbers could not be paired. Signal an
> error if all complex numbers are not exact conjugates (to within
> tol). Note that there is no defined order for pairs with identical
> real parts but differing imaginary parts.
>
> ```
>           cplxpair (exp(2i*pi*[0:4]'/5)) == exp(2i*pi*[3; 2; 4; 1; 0]/5)
> ```

— Mapping Function: **imag** (z)  
> Return the imaginary part of z as a real number.
>
>
>
> **See also:** real, conj.

— Mapping Function: **real** (z)  
> Return the real part of z.
>
>
>
> **See also:** imag, conj.
